# Therapeutic Effects of Dihydromyricetin on Wholly Alcohol-Attributed Conditions: A Systematic Review

**DOI:** 10.3390/nu18142221

**Published:** 2026-07-08

**Authors:** Samantha G. Skinner, Saikumar Matcha, Daryl L. Davies

**Affiliations:** Titus Family Department of Clinical Pharmacy, Alfred E. Mann School of Pharmacy and Pharmaceutical Sciences, University of Southern California, Los Angeles, CA 90033, USA; sgskinne@usc.edu (S.G.S.); matcha@usc.edu (S.M.)

**Keywords:** dihydromyricetin, alcohol, alcohol-associated liver disease, alcohol use disorder

## Abstract

**Background**: Alcohol use is a major global health burden and is causally linked to several wholly alcohol-attributed conditions, including alcohol use disorder (AUD) and alcohol-associated liver disease (ALD). Current therapeutic options remain limited. Dihydromyricetin (DHM), a plant-derived flavonoid with antioxidant and anti-inflammatory properties, has emerged as a potential candidate for mitigating alcohol-induced toxicity. This systematic review aimed to comprehensively evaluate the therapeutic effects of DHM across alcohol-related conditions. **Methods**: A systematic literature search was conducted in PubMed from inception through December 2025 for studies investigating the effects of DHM or DHM-containing extracts on alcohol-related outcomes. Both preclinical (in vitro and in vivo) and clinical studies were considered. Study quality was assessed qualitatively due to heterogeneity precluding use of a standardized risk-of-bias tool. Results were synthesized narratively by outcome category; meta-analysis was not performed. This review was unregistered with no prior protocol. **Results**: A total of 22 studies were included, comprising 8 in vitro, 17 in vivo, and 2 clinical studies, with some studies contributing data to more than one category. Across models, DHM consistently attenuated ethanol-induced cytotoxicity, oxidative stress, inflammation, and hepatic steatosis. DHM improved liver injury biomarkers (e.g., AST and ALT), enhanced antioxidant defenses, and modulated key signaling pathways including Nrf2 and AMPK. Additionally, DHM supported mitochondrial function and intestinal barrier integrity. However, findings related to ethanol metabolism and neurobehavioral outcomes were inconsistent. Clinical evidence was limited to two small trials using *Hovenia dulcis* extracts, which demonstrated reductions in hangover severity and selected inflammatory markers but did not directly evaluate isolated DHM. **Conclusions**: DHM demonstrates robust preclinical efficacy in mitigating alcohol-induced injury, particularly in hepatic outcomes. Despite promising mechanistic and experimental evidence, clinical data remain limited. The certainty of evidence is constrained by preclinical study heterogeneity, the absence of formal risk-of-bias assessment, and the lack of clinical trials using isolated DHM. Well-designed clinical trials using standardized DHM formulations are needed to establish its complete therapeutic potential in alcohol-related disorders.

## 1. Introduction

Alcohol (ethanol/EtOH) remains the most widely consumed drug of abuse globally and continues to be a major contributor to preventable morbidity and mortality [1]. Alcohol consumption is one of the leading risk factors for global mortality, ranking eighth overall [2,3]. In 2019, alcohol use was responsible for an estimated 2.6 million deaths worldwide, reflecting its substantial burden on public health [1]. Alcohol consumption has been causally linked to more than 200 diseases, injuries, and health conditions, ranging from acute harms to chronic noncommunicable diseases [1,4]. While alcohol acts as a contributing factor in many of these conditions, several diseases are classified as wholly alcohol-attributed conditions [5]. These conditions are readily identifiable by their diagnostic definitions, which explicitly incorporate alcohol, and include alcohol use disorder (AUD), alcohol-associated liver disease (ALD), and fetal alcohol spectrum disorders (FASDs).

AUD is a chronic relapsing condition characterized by compulsive alcohol use, impaired control over intake, and the emergence of tolerance and withdrawal symptoms [6,7]. The global burden of AUD is substantial. Around 400 million individuals aged 15 years and older, i.e., 7% of the world’s adult population, were living with AUD in 2019 [1]. In the United States, alcohol use remains highly prevalent, with approximately 62% of adults reporting past year consumption in 2024 [8] and ~10% of individuals meeting criteria for AUD [9]. Notably, up to 95% of individuals with an AUD exhibit some degree of ALD pathology [7].

ALD represents a progressive spectrum of liver injury, ranging from steatosis to steatohepatitis, and cirrhosis [10]. In recent years, the prevalence of ALD has grown such that nearly half of all liver disease-related mortality can be attributed to alcohol [11]. Furthermore, ALD has surpassed hepatitis C virus infection as the leading cause of liver transplantation due to chronic liver disease [12]. The pathogenesis of ALD is primarily driven by hepatic ethanol metabolism, a highly reactive process that generates damaging byproducts such as acetaldehyde and reactive oxygen species (ROS) [13]. These metabolites disrupt cellular homeostasis through protein and DNA adduct formation, the induction of mitochondrial stress, and the promotion of lipid peroxidation, ultimately promoting inflammation and tissue injury [14,15]. Beyond its effects in adults, alcohol exposure during pregnancy may result in fetal alcohol spectrum disorders (FASDs), which encompass a range of developmental abnormalities including neurobehavioral deficits of varying severity [16].

Collectively, the clinical and economic burden of alcohol use is substantial, accounting for over four million emergency room visits and an estimated economic cost of $249 billion annually [17]. Despite the growing disease burden, therapeutic options remain limited. Currently only three medications —disulfiram, acamprosate, and naltrexone—are approved by the United States Food and Drug Administration (FDA) for the treatment of AUD [18]. These drugs demonstrate only modest efficacy in reducing alcohol consumption and maintaining abstinence, and their clinical impact is further constrained by low prescription rates, with only ~2% of individuals with AUD receiving medication [17,19]. Currently, there are no disease modifying, targeted pharmacotherapies specifically approved for ALD. Instead, disease management is largely limited to corticosteroids for severe alcoholic hepatitis and liver transplantation in advanced cases [20]. Similarly, treatment options for FASDs are primarily supportive and symptom based. These limitations highlight a critical unmet need for novel therapeutic strategies targeting wholly alcohol-attributable conditions. Because oxidative stress and inflammation are central drivers of ethanol-induced hepatic and neural injury, compounds with multi-target antioxidant and anti-inflammatory activity represent particularly attractive candidates for intervention.

Dihydromyricetin (DHM) is a dihydroflavonol (a flavonoid subclass, also termed a flavanonol) isolated from several plant species, including *Ampelopsis grossedentata* and *Hovenia dulcis*, that has gained increasing attention due to its antioxidant and anti-inflammatory properties [21,22]. The therapeutic use of this flavonoid traces back to China’s first pharmacopeia, Xinxiu Bencao, which designated *Hovenia dulcis* as an anti-hangover remedy. Today, this herbal compound is readily available as an over-the-counter nutraceutical supplement and marketed for its alcohol hangover relieving effects, reflecting longstanding cultural and commercial interest in its potential anti-alcohol effects. Although DHM has been investigated across multiple experimental contexts, its efficacy in alcohol-related conditions has not been systematically synthesized. Therefore, the aim of this systemic review is to critically assess the effect of DHM on alcohol-related outcomes by integrating evidence from in vitro, in vivo, and clinical studies, with the goal of clarifying its therapeutic potential.

## 2. Materials and Methods

This systematic review was guided by the Preferred Reporting Items for Systematic Reviews and Meta-Analyses (PRISMA) 2020 reporting principles [23], with deviations noted where applicable, including the use of a qualitative rather than a formal risk-of-bias assessment given the heterogeneity of included study designs. The review was not prospectively registered, and no protocol was prepared prior to conduct.

### 2.1. Search Strategy

A systematic literature search was conducted in PubMed from inception to December 2025. The search was limited to PubMed, as it represents the primary database for biomedical literature relevant to the scope of this review. The following search string was applied: (“Dihydromyricetin” OR “*Ampelopsis grossedentata*” OR “*Hovenia dulcis*” OR “Flavonoids” OR “Ampelopsis” AND “Alcohol” OR “Alcohol-induced toxicity” OR “Alcohol use disorder” OR “Alcohol-associated liver disease” OR “Alcohol Induced disorders” OR “Ethanol” OR “Alcohol-related disorders” OR “hangover”). This search string enabled the identification of studies investigating the effects of DHM on alcohol-related outcomes. In addition, relevant articles were manually screened from reference lists of selected publications. All study designs were considered, including preclinical and clinical studies, to provide a comprehensive overview of DHM effects.

### 2.2. Study Selection Criteria

Original research studies that directly reported DHM or DHM-containing extract effects on alcohol-related outcomes were included. Studies were excluded if DHM was administered in combination with other drugs and the independent effects of DHM could not be determined. The study selection process followed PRISMA guidelines, including title and abstract screening, full-text review, and documentation for reasons for exclusion of articles at each stage. Two independent authors (S.G.S. and S.M.) individually screened the publications, and any discrepancies were resolved with the assistance of D.L.D.

### 2.3. Data Extraction

Relevant data from each included study were systematically extracted using a predesigned summary sheet. Extracted information included study design (in vitro, in vivo, or clinical), species or cell type, experimental methods (including diet or treatment protocols), DHM dose and route of administration, ethanol exposure or alcohol-induced toxicity model, outcome measures, observed effects of DHM, and key findings. This standardized approach ensured consistency and facilitated comparative analysis across different study types.

### 2.4. Risk Assessment

Due to substantial heterogeneity in study designs (including in vitro, in vivo, and clinical studies) and outcome measures, a single standardized risk-of-bias assessment was not appropriate for all included studies. Instead, study quality was evaluated qualitatively by examining key methodological features, including study design, sample size, use of control groups, reporting of randomization and blinding procedures where applicable, intervention characteristics, and outcome reporting. These characteristics are summarized in the tables to facilitate transparent comparison and interpretation of the evidence base. Given this heterogeneity, meta-analysis was not performed. In the absence of meta-analysis, findings are reported as direction of effect and statistical significance as described by the original study authors. Results were synthesized narratively and organized by outcome category. No sensitivity analyses or formal assessment of publication bias or certainty of evidence were conducted.

## 3. Results

### 3.1. Overview of Included Studies

**Figure 1:**
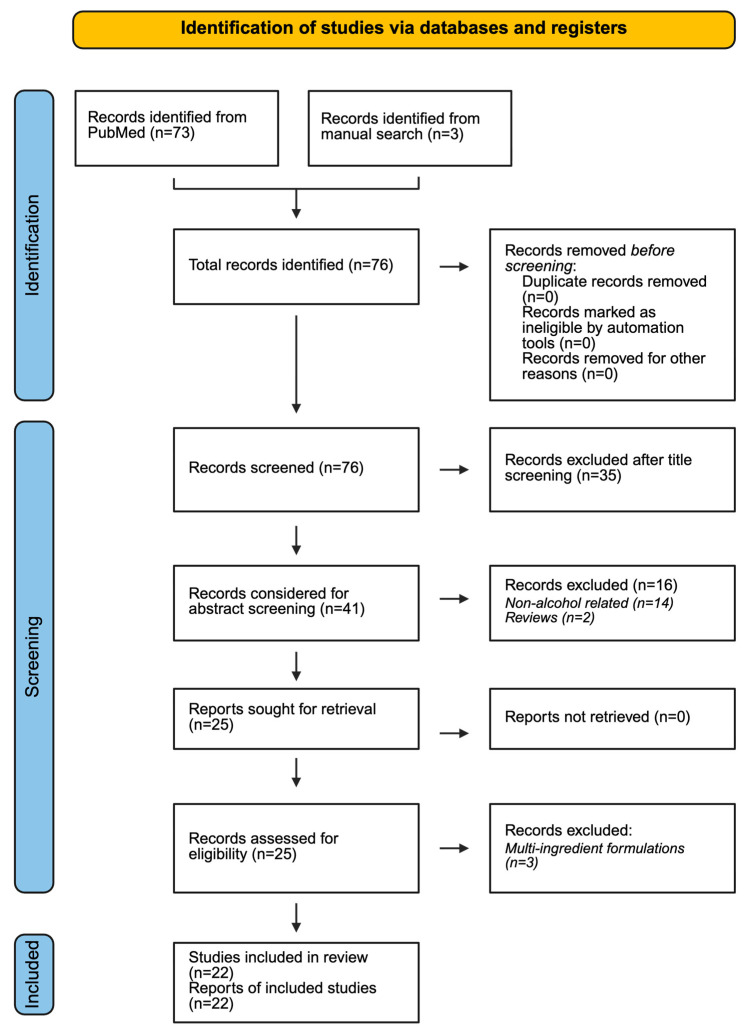
Prisma flowchart.

A total of 76 records, including 3 manually identified articles, were retrieved through the literature search. Following title and abstract screening, 25 articles were selected for full-text review. Three studies were excluded following full-text review because DHM was administered as part of a multi-ingredient formulation, therefore making the determination of independent anti-alcohol effects impossible [24,25,26]. Of these, 22 studies were ultimately included in the systematic review. The screening procedure and results at each stage are illustrated in Figure 1.

Of the included studies, 8 reported in vitro findings, 17 reported in vivo findings, and 2 reported clinical data. Some studies contributed findings across multiple categories; therefore, category totals exceed the number of unique included studies. Detailed characteristics of the included in vitro and in vivo studies, including DHM or DHM-containing extract dosage and EtOH administration model, are, respectively, summarized in Table 1 and Table 2 to facilitate comparison across heterogeneous study designs.

### 3.2. In Vitro Effects on Cell Culture Models

#### 3.2.1. Cell Viability and Cytotoxicity

Multiple in vitro studies demonstrate that DHM protects against alcohol-induced cytotoxicity across a range of cell types, with the most extensive evidence derived from hepatocyte models. In Alpha Mouse Liver 12 (AML-12) murine hepatocytes, both concurrent DHM treatment and DHM pre-treatment significantly improved cell proliferation compared with EtOH treatment alone across a range of low-micromolar DHM concentrations (approximately 0.1–100 µM; Table 1) [27,30]. DHM pre-treatment also reduced lactate dehydrogenase (LDH) release relative to EtOH-treated cells, indicating preservation of membrane integrity [30]. These findings were consistent in human hepatoblastoma-derived hepatocytes, where treatment with DHM significantly attenuated cytotoxicity induced by EtOH [32].

Protective effects of DHM on cell viability were also observed in non-hepatic cell types. In intestinal epithelial cells, DHM pre-treatment significantly improved cell viability following ethanol exposure, with effects exceeding those observed for other polyphenols including chlorogenic acid, apigenin, and phloretin [30]. In SH-SY5Y human neuroblastoma cells, DHM conferred concentration-dependent protection against ethanol-induced toxicity, with complete protection observed at 0.1 μM [31]. Importantly, DHM alone did not affect cell viability at any tested concentration, indicating a lack of intrinsic cytotoxicity.

#### 3.2.2. Oxidative Stress and Reactive Oxygen Species

Multiple studies demonstrate that DHM mitigates alcohol-induced oxidative stress through both direct antioxidant activity and modulation of endogenous cellular defense pathways, the latter primarily via activation of the Keap1-Nrf2 pathway (described below). In hepatocyte models, DHM or *Ampelopsis grossedentata* extract (AGE) treatment significantly reduced EtOH-generated intracellular ROS in both ethanol-oxidizing (VL-17A) and non-oxidizing (HepG2) human hepatocellular carcinoma cell lines, as well as transformed human liver epithelial 2 (THLE-2) cells [28,32]. In primary rat hepatocytes, DHM treatment similarly reduced levels of both ROS and reactive nitrogen species [33]. Consistent protective effects were observed in AML-12 murine hepatocytes, where both concurrent administration of DHM with EtOH and DHM pre-treatment significantly attenuated alcohol-induced ROS accumulation. In this model, DHM also restored depleted antioxidant defenses, including superoxide dismutase (SOD), catalase, and glutathione (GSH), and reduced lipid peroxidation, as evidenced by decreased malondialdehyde (MDA) levels [27,30]. Consistent with these results, Western blot analyses demonstrated increased catalase expression in both HepG2 and VL-17A cells following DHM treatment [32]. Additionally, AGE administration led to the restoration of peroxisome proliferator-activated receptor gamma coactivator-1α (PGC-1α) and sirtuin (SIRT)-3 expression following EtOH-induced downregulation [28].

Antioxidant and antigenotoxic effects of *Hovenia dulcis* extracts (HDE) against ethanol-induced damage were also examined in SH-SY5Y human neuroblastoma cells, where pre-treatment with HDE prior to EtOH exposure significantly reduced DNA strand breaks and superoxide anion production [29]. Importantly, these findings were observed in the absence of DHM as an isolated compound; DHM was not identified in the HPLC characterization of these extracts. The observed effects may therefore be attributable to other *Hovenia dulcis* constituents rather than to DHM.

#### 3.2.3. Lipid Metabolism and Steatosis

DHM treatment significantly attenuated alcohol-induced lipid accumulation across multiple hepatocyte models. In both HepG2 and VL-17A cells, EtOH resulted in marked increases in intracellular lipid content, while DHM co-treatment significantly reduced this accumulation in a dose-dependent manner [32]. This effect was also observed in AML-12 cells; however, this protection was partially reversed when cells were transfected with microRNA-155-5p (miR-155-5p) mimics [27].

DHM also affects key regulators of lipogenesis and fatty acid oxidation. In HepG2 and VL-17A cells, DHM treatment significantly reduced levels of the lipogenic transcription factor sterol regulatory element-binding protein-1 (SREBP-1) during ethanol exposure [32]. Concurrently, DHM activated the AMP-activated protein kinase (AMPK) pathway, as evidenced by increased phosphorylation of AMPK and its downstream target acetyl-CoA carboxylase-1, which is consistent with reduced lipogenesis and enhanced fatty acid oxidation [32]. Furthermore, both DHM and DHM-containing extract treatment increased expression of carnitine palmitoyltransferase-1a (CPT1a), a key regulator of mitochondrial fatty acid transport [32,40].

#### 3.2.4. Ethanol and Acetaldehyde Metabolism—*In Vitro*

DHM demonstrates the capacity to enhance ethanol and acetaldehyde metabolism in hepatocyte models, though findings are inconsistent. In vitro metabolic studies demonstrated that DHM enhanced ethanol and acetaldehyde clearance in both HepG2 and VL-17A cells. Additionally, in VL-17A cells, DHM significantly increased acetic acid production, indicating enhanced metabolic conversion through the complete pathway. Consistent with these functional outcomes, Western blot analysis revealed that DHM increased protein expression of alcohol dehydrogenase (ADH) 1 and aldehyde dehydrogenase (ALDH) 2 in these cell lines, suggesting enhanced metabolic capacity [32]. In addition to effects on classical ethanol-metabolizing enzymes, DHM pre-treatment reduced messenger RNA (mRNA) and protein expression of cytochrome P450 2E1 (CYP2E1) in both AML-12 hepatocytes and intestinal epithelial cell line 6 (IEC-6) [30]. Molecular docking analyses further predicted that DHM forms hydrogen bonds with CYP2E1 amino acid residues, suggesting a potential direct interaction. However, contrasting results were observed in primary rat hepatocytes, where DHM did not significantly modulate ADH or CYP2E1 activity [33]. The authors therefore concluded that DHM’s hepatoprotective effects in their system could not be attributed to altered ethanol metabolic enzyme activity.

### 3.3. In Vivo Effects in Animal Models

In vivo studies were performed in mice and rat models. Ethanol effects were evaluated using multiple different exposure paradigms, including acute, chronic, and binge protocols, which involved either single or repeated bolus ethanol gavages [33,35,40,41,44,46], single ethanol injections [32,34], two-bottle choice procedure [34], single- bottle access [32,42], or chronic administration via the Lieber–DeCarli diet [27,28,36,38,40,45]. DHM treatments were administered either concurrently with ethanol exposure or prior to ethanol induction, at doses ranging from 5 mg/kg to 2 g/kg via oral (p.o.) or intraperitoneal (i.p.) routes. Most studies included control, ethanol, and DHM treatment groups, while some experiments were limited to ethanol and DHM arms only.

#### 3.3.1. Biochemical Markers of Alcohol-Induced Liver Injury

Aspartate Aminotransferase (AST) and Alanine Aminotransferase (ALT) are routinely assessed serum or plasma biomarkers of liver function. In preclinical studies, both markers were significantly elevated in the ethanol-treated groups compared to controls. Treatment with DHM or DHM-containing extracts restored these biomarkers towards normal levels, with most studies reporting statistically significant reductions compared to the ethanol group [27,28,32,36,38,40,44,45]. However, one study reported that native DHM did not significantly reduce serum ALT or AST relative to the ethanol-treated group; in contrast, a micellar formulation of DHM (termed DMY-Ms by authors), prepared using Solutol^®^HS15 to enhance oral bioavailability, produced significant reductions in both markers at the same dose [44].

Alkaline phosphatase (ALP) and lactate dehydrogenase (LDH) were assessed in a subset of studies [27,28,36,45]. Both markers were significantly elevated in ethanol-treated groups relative to controls, and DHM or DHM-containing extract treatment significantly attenuated their serum levels in all four studies. Additionally, treatment with DHM or DHM-containing extract significantly attenuated alcohol-induced increases in liver weight [27,32]. This reduction in liver weight likely parallels the observed decrease in hepatic lipid accumulation (steatosis), although concurrent reductions in inflammation and associated edema may also contribute. Serum triglyceride levels were consistently decreased by DHM or AGE across studies [32,38,40]. DHM treatment also affected other lipid biomarkers: free cholesterol was significantly reduced, and low-density lipoprotein/very low-density lipoprotein (LDL/VLDL) showed a trend toward decrease, while cholesteryl esters were nonsignificantly increased [38].

#### 3.3.2. Hepatic Histopathology and Steatosis

Hepatic histopathological analysis revealed that chronic EtOH administration induced marked steatosis and liver injury, whereas these pathological changes were markedly reversed by both DHM and AGE treatment [27,28,32,36,38,40,45]. Specifically, Hematoxylin and Eosin (H&E) staining demonstrated marked disruption of hepatic architecture, characterized by extensive hepatocyte ballooning, and inflammatory cell infiltration. In contrast, mice treated with DHM displayed substantial improvement in hepatic morphology, evidenced by preserved lobular architecture, reduced hepatocyte ballooning, and less inflammatory cell infiltration [27,28,36,38,40,45].

Oil Red O staining, which was used to evaluate lipid deposition within hepatocytes, revealed that ethanol-treated mice exhibited intense punctate staining, reflecting excessive accumulation of intracellular lipid droplets. In comparison, DHM- and AGE-treated mice showed a pronounced reduction in lipid accumulation, with fewer lipid droplets visible [27,28,32,36,38,45]. This reduction in lipid droplets was highlighted as dose dependent in one study [27]. While most studies histology suggests decreased lipid droplet size with DHM, Janilkarn-Urena et al. [38] observed that while DHM reduced the total number of lipid droplets, treatment led to increased individual droplet size as well as noticeable hepatocyte-to-hepatocyte lipid droplet heterogeneity.

Biochemical analysis further supported these observations. Hepatic triglyceride levels were significantly elevated in ethanol-treated animals, reflecting excessive lipid storage in the liver. DHM or AGE administration reduced hepatic triglyceride content in most studies [28,32,40], demonstrating efficacy in attenuating ethanol-induced lipid accumulation. Notably, one study reported increased hepatic triglycerides with DHM treatment, though circulating triglycerides were reduced [38].

#### 3.3.3. Oxidative Stress and Antioxidant Defense

Several oxidative stress biomarkers were measured in vivo, including GSH, SOD, and MDA. Both DHM and AGE consistently reduced lipid peroxidation in the liver and stomach, as evidenced by decreased MDA levels [36,39,44,45]. Similarly, 4-Hydroxynonenal (4-HNE), another marker of lipid peroxidation and oxidative injury, was significantly decreased following DHM administration [32]. Studies examining alternative DHM formulations, including DMY-Ms and DHM-SEDDS, also demonstrated significant reductions in MDA following acute ethanol exposure [39,44].

In parallel with reductions in oxidative damage, antioxidant capacity was significantly enhanced in the liver and stomach with DHM. GSH levels increased following treatment with both DHM and AGE [36,45], while SOD2 expression significantly increased with AGE administration [28]. The alternative DHM formulations (DMY-Ms and DHM-SEDDS) also produced significant increases in SOD activity after acute ethanol administration [39,44]. Furthermore, both DHM and AGE activated the Nrf2 signaling pathway in the liver, producing dose-dependent increases in nuclear factor erythroid 2-related factor 2 (Nrf2) expression, which was accompanied by dose-dependent increases in heme oxygenase-1 (HO-1) expression as well as decreased Keap-1 expression, reversing the ethanol-induced pattern of elevated Keap-1 and reduced HO-1 [32,45]. These findings were confirmed by Western blot analysis, with Keap-1 results further supported by immunohistochemistry.

#### 3.3.4. Mitochondrial Function and Biogenesis

In addition to modulating oxidative stress and antioxidant signaling, DHM also influences hepatic mitochondrial function and biogenesis. Chronic alcohol intake elevated mitochondrial protein markers voltage-dependent anion channel 1 (VDAC1) and translocase of the outer mitochondrial membrane 20 (TOMM20), suggesting altered mitochondrial mass or turnover; these changes were markedly reversed by DHM treatment [27]. Functionally, ethanol reduced hepatic ATP concentrations, mitochondrial DNA (mtDNA)/nuclear DNA (nDNA), and the NAD^+^/NADH ratio, all of which were restored by DHM administration [42]. This was also true of mtDNA copy number, which DHM normalized in a dose-dependent manner [27]. DHM administration also further enhanced the expression mitochondrial transcription factor A (TFAM), a key regulator of mtDNA transcription and replication [42].

Administration of both DHM and DHM-containing extract also regulated the PGC-1α/SIRT3 pathway, a critical regulator of mitochondrial biogenesis, which is significantly impaired by ethanol. In the liver, alcohol decreased both mRNA and protein levels of PGC-1α, increased PGC-1α acetylation, and suppressed SIRT3, SIRT1, and pAMPK expression as well as overall SIRT deacetylase activity; these alterations were reversed by DHM or AGE treatment [28,42]. DHM also attenuated the expression of YTH N6-methyladenosine RNA binding protein 2 (YTHDF2), an RNA binding protein that mediates PGC-1α/SIRT3 expression dysregulation [28].

Furthermore, mitochondrial respiratory chain function was assessed through both protein expression and oxidative phosphorylation complex activity measurements. Ethanol reduced Complex I activity, which was significantly restored by AGE or DHM [28,38]. For Complex II and Complex IV, ethanol reduced their activities, and DHM restored function to baseline levels [38]. Additionally, ethanol decreased expression of mitochondrial Complex III and Complex V, while DHM increased expression of both complex proteins [42].

#### 3.3.5. Intestinal Barrier Function and Gut-Liver Axis

EtOH is well known to impact the gastrointestinal tract, contributing to mucosal injury and increased intestinal permeability. In the stomach, DHM treatment reduced gastric mucosal injury (i.e., bleeding and ulceration), with enhanced protection observed with DHM-SEDDS or DMY-Ms formulations [39,44]. This protection extends to the small intestine. Histological evaluation of duodenal tissue by H&E staining revealed severe mucosal injury in the EtOH group. In contrast, the DHM-treated group exhibited restored villous architecture [35].

Additionally, AGE treatment restored ethanol-induced reductions in duodenal and ileal tight junction proteins occludin and zonulin-1 (ZO-1), as well as the mucus layer component mucin-2, indicating improved epithelial barrier integrity and mucosal protection [35,36]. Consistent with these findings, in vitro studies demonstrated that DHM upregulated occludin, ZO-1, and claudin-1 expression in intestinal epithelial cells, further supporting its role in preserving barrier function [30].

Functionally, EtOH significantly increases circulating levels of lipopolysaccharide; AGE significantly reduced these levels to baseline [36]. Additionally, AGE pre-treatment improved ethanol-induced abnormalities in duodenal amino acid metabolism, suggesting broader restoration of intestinal metabolic homeostasis [35].

#### 3.3.6. Inflammatory Markers and Immune Response

Chronic ethanol exposure robustly activates inflammatory pathways, characterized by increased pro-inflammatory cytokine production and innate immune signaling. Across multiple studies, ethanol significantly elevated hepatic and serum levels of tumor necrosis factor alpha (TNF-α), interleukin (IL)-1β, IL-6, IL-1α, IL-17, and interferon (IFN) -γ, as well as circulating chemokines, including CXCL5 and CXCL10 [27,28,32,35,36,38,45]. DHM or AGE treatment consistently attenuated these elevations. In addition to suppressing pro-inflammatory mediators, DHM increased the expression of anti-inflammatory cytokines in circulation including IL-1ra and IL-27α, suggesting a broad immunomodulatory effect [38].

Ethanol-induced inflammation is closely associated with activation of inflammatory signaling pathway proteins. Ethanol exposure resulted in increased expression of cluster of differentiation (CD) 14, Toll-like receptor (TLR) 4, TLR9, myeloid differentiation primary response (MyD) 88, and nuclear factor kappa B (NF-κB), accompanied by elevated phosphorylation of inhibitor of κB alpha (IκB-α), indicative of NF-κB activation [27,36,45]. Both DHM and AGE administration reversed these changes, reducing TLR/MyD88 signaling and normalizing the p-IκB-α/IκB-α ratio, thereby suppressing NF-κB-mediated transcription of inflammatory genes. In parallel, AGE attenuated ethanol-activated mitogen-activated protein kinase (MAPK) signaling cascades, as demonstrated by restored phosphorylated-to-total protein ratios of c-Jun N-terminal kinase (JNK), extracellular signal–regulated kinase (ERK), and MAPK p38 [35].

DHM supplementation also reduced ethanol-induced immune cell infiltration, with diminished monocyte/macrophage accumulation in hepatic tissue compared to ethanol-exposed mice [38]. Consistent with these findings, DHM lowered levels of hematopoietic cytokines and chemokines elevated during chronic alcohol consumption, including granulocyte–macrophage colony-stimulating factor (GM-CSF), macrophage colony-stimulating factor (M-CSF), granulocyte (G)-CSF, IL-3, CXCL1, CXCL2, and CXCL13, as well as additional inflammatory mediators such as TIMP1, ICAM-1, CCL1, CCL9, CCL21, DPP-4, CRP, and C5a [36,38].

#### 3.3.7. Ethanol and Acetaldehyde Metabolism

Reported effects of DHM on ethanol metabolism are variable. Treatment with DHM or DHM-containing extract led to a significant reduction in blood ethanol concentrations (BEC), reflecting enhanced ethanol clearance [34,37,46]. Correspondingly, serum EtOH and acetaldehyde concentrations were reduced in DHM-treated groups [32]. This effect was accompanied by a marked increase in cytosolic ADH as well as cytosolic and mitochondrial ALDH expression and activity in the liver, promoting the efficient conversion of EtOH [32,37]. Additionally, ethanol-induced upregulation of CYP2E1 was attenuated by DHM treatment [32,40].

However, these findings are not consistent across studies. Wu et al. observed no significant reduction in BEC, despite a downward trend, and reported no significant changes in ALDH2 expression [40]. Similarly, Skotnicova et al. found that DHM did not significantly alter BEC or acetaldehyde concentrations [33]. Ye et al. reported only minor changes in BEC following native DHM administration, whereas DMY-Ms produced more substantial reductions [44]. Likewise, Dong et al. found that native DHM did not significantly affect serum EtOH concentrations or ADH and ALDH2 activity in hepatic or gastric tissues, while a self-emulsifying drug delivery system (DHM-SEDDS) significantly enhanced enzyme activity [39].

#### 3.3.8. Neurobehavioral and Functional Outcomes

Evidence regarding the effects of DHM on neurobehavioral outcomes also remains variable. Several studies report that DHM attenuates ethanol-induced intoxication, as shown by a time-dependent reduction in loss of righting reflex (LORR) in both male and female mice [34,41]. However, other work found no change in LORR following administration of native DHM; in those cases, only enhanced formulations (e.g., DHM-SEDDS or DMY-Ms) produced significant improvements, such as faster recovery of the righting reflex [39,44]. Similarly, although DHM has been reported to prevent escalation of alcohol consumption and to reduce established high intake in a chronic alcohol consumption model [34], some studies observed no effect of DHM alone on ethanol preference or consumption in either sex [43]. Interestingly, DHM did reduce alcohol intake when co-administered with ivermectin [43].

DHM also appears to influence withdrawal behavior. Single EtOH exposure produced tolerance to subsequent EtOH challenge; DHM co-administration prevented this tolerance development [34]. EtOH exposure/withdrawal produced anxiety-like behavior; DHM co-administration prevented this withdrawal-induced anxiety without affecting baseline anxiety [34]. EtOH exposure/withdrawal increased pentylenetetrazol (PTZ)-induced seizure susceptibility and CNS hyperexcitability; DHM ameliorated these effects [34].

Although limited, evidence from a developmental model suggests that DHM may confer protective effects during prenatal ethanol exposure. Co-administration of DHM with ethanol in pregnant rats prevented fetal alcohol exposure-induced behavioral and physiological abnormalities in offspring, normalizing anxiety-like behavior, anesthetic sensitivity to acute ethanol, and PTZ-induced seizure susceptibility compared to offspring of ethanol-treated dams [46]. Furthermore, DHM during prenatal ethanol exposure maintained both synaptic and extrasynaptic gamma-aminobutyric acid A receptor (GABA_A_R) sensitivity, indicating protection of inhibitory signaling in the developing brain [46]. Given the absence of disease-modifying treatments for FASDs, the preclinical evidence that DHM attenuates prenatal ethanol-induced abnormalities is especially noteworthy and warrants prioritization in future work.

### 3.4. Clinical Evidence

Two clinical studies evaluated the effects of DHM-containing extracts on alcohol-related outcomes, namely hangover, in humans [47,48]. In the first study, conducted in 26 healthy male adults, participants were assigned to receive Korean soju (50 g alcohol) in combination with either HDE (2460 mg) or placebo. Blood alcohol concentration (BAC), acetaldehyde levels, and total hangover scores peaked at 1 h post-administration, with no significant differences observed between groups at this time point. However, a significantly greater reduction in total hangover scores was observed in the HDE group at 4 and 12 h compared with placebo. Serum biomarkers of gut permeability and endotoxemia, including endotoxin, lipopolysaccharide-binding protein (LBP), and soluble CD14, did not differ between groups at 0, 4, and 12 h. In contrast, inflammatory markers were affected: TNF-α and IL-6 levels were lower in the HDE group, with IL-6 showing statistically significant differences at 4 and 12 h. Additionally, AST levels increased significantly over 12 h in the placebo group, whereas a significant decrease from baseline was observed in the HDE group [47].

The second study included 25 adult participants in a randomized, double-blind, placebo-controlled, parallel-group design. Participants received beverages containing HDE combined with either *Pueraria lobata* extract (HDPB) or glutathione yeast extract (HDGB), or placebo. All formulations were administered as 500 mL beverages. HDPB and HDGB formulations contained 0.731% HDE, with differences in additives: HDPB included 0.1% (*v*/*v*) *Pueraria lobata* extract, whereas HDGB contained 0.02% (*w*/*v*) glutathione yeast extract. Both HDPB and HDGB groups demonstrated significantly lower Alcohol Hangover Scale (AHS) gastrointestinal scores compared to the placebo. Blood alcohol concentrations were reduced in the HDPB and HDGB groups within the first 30 min post-consumption, consistent with preclinical evidence suggesting that DHM may delay alcohol absorption. Notably, higher acetaldehyde levels were observed in these groups, which the authors attributed to potentially reduced ALDH activity and slower acetaldehyde clearance [48]. Importantly, the above studies did not evaluate isolated DHM. Therefore, the clinical findings cannot be directly attributed to DHM alone.

## 4. Discussion

This systematic review synthesizes evidence across 22 studies investigating the therapeutic effects of DHM for wholly alcohol-attributed conditions. Across models, DHM consistently demonstrated protective effects against alcohol-induced injury. The most consistent findings were DHM’s attenuation of ethanol-induced cytotoxicity, reduction in hepatic steatosis, normalization of serum liver injury markers, suppression of oxidative stress, and attenuation of pro-inflammatory cytokine signaling. Evidence also supports that DHM enhances mitochondrial function and preserves intestinal barrier integrity, although these findings are derived from a smaller number of studies and warrant further investigation. In contrast, preclinical evidence for DHM’s effects on ethanol metabolism and neurobehavioral outcomes, including BEC, intoxication, and voluntary alcohol consumption, were inconsistent across studies, with positive and null findings. Additionally, two clinical trials with small sample sizes demonstrated that HDE reduced hangover severity and attenuated select alcohol-associated inflammatory markers. Taken together, the available evidence provides meaningful preclinical support for DHM as a potential therapeutic candidate in wholly alcohol-attributed conditions, particularly ALD, though the absence of clinical data for AUD or ALD represents a critical gap.

Several mechanistic pathways emerged across the included studies. In vitro evidence suggests DHM’s antioxidant effects appear to be mediated, in part, through activation of the Keap1-Nrf2 pathway, as DHM suppressed Keap1 expression and promoted nuclear translocation of phosphorylated Nrf2, increasing downstream antioxidant enzyme expression [30]. Molecular docking analyses further predicted in silico that DHM binds directly within the Keap1 pocket, potentially disrupting the Keap1-Nrf2 interaction and facilitating Nrf2 activation, with DHM demonstrating the strongest predicted binding affinity among the four polyphenols examined in that study [30]. Additional reported hepatic mechanisms include modulation of the miR-155-5p/SIRT1/VDAC1 pathway, which may restore Kupffer cell–hepatocyte communication under chronic alcohol exposure by reducing inflammation and senescence [27]. At the level of metabolic signaling, Silva et al. demonstrated that DHM activates AMPK-dependent signaling pathways, including the AMPK/SIRT1/PGC-1α axis, leading to enhanced lipid oxidation, improved mitochondrial function, and restoration of metabolic homeostasis following ethanol exposure [42]. Whether DHM activates AMPK directly or indirectly (for example, secondary to changes in the AMP/ATP ratio or to SIRT1-mediated signaling) was not established in the included studies and remains to be clarified. Consistent with this upstream signaling, Janilkarn-Urena et al. showed that DHM enhances lipophagy by promoting the colocalization of autophagy proteins at lipid droplets, facilitating their clearance and reducing ethanol-induced lipotoxicity [38]. That being said, a direct causal relationship between AMPK activation and this lipophagic response was not formally established. In the CNS, Shen et al. identified GABA_A_Rs as a direct molecular target of DHM, demonstrating that DHM acts as a positive modulator at the benzodiazepine binding site of GABA_A_Rs, evidenced by increased GABA responses and competitive inhibition of benzodiazepine ligand binding. Critically, DHM also prevented ethanol exposure/withdrawal-induced GABA_A_R plasticity through this same mechanism, suggesting a potential basis for its observed effects on tolerance and withdrawal [34]. Collectively, these findings indicate that DHM exerts protective effects in both the liver and CNS through multiple, potentially complementary mechanisms, though the relative contributions of these pathways remain to be fully elucidated.

To our knowledge, this is the first systematic review to comprehensively synthesize the evidence for DHM’s anti-alcohol effects. Strengths of the study include the assessment of evidence across in vitro, in vivo, and clinical study designs, spanning multiple alcohol-related conditions and outcome domains, as well as consistency with PRISMA 2020 reporting principles using a standardized data extraction process. Nonetheless, several limitations should be acknowledged. First, due to the inclusion of diverse study designs, a formal risk of bias assessment was not conducted, limiting the ability to evaluate study quality systematically. Furthermore, reporting of methodological safeguards commonly used to reduce risk of bias in preclinical studies, including randomization, blinding, and sample size justification, was inconsistent across the included literature, limiting the ability to systematically evaluate study quality. Second, the substantial methodological heterogeneity across studies complicates the interpretation of study findings. Third, literature retrieval was restricted to PubMed. However, to enhance search completeness, reference lists of all included studies and relevant review articles were manually screened, resulting in the identification of three additional eligible studies that were not retrieved through the primary database search. Although this supplementary approach helped reduce the likelihood of missing relevant literature, studies indexed exclusively in other databases may still have been overlooked.

This heterogeneity is observed across multiple dimensions. DHM doses varied across an approximately 6000-fold range in in vivo studies (0.3 mg/kg to 2 g/kg), routes of administration included both intraperitoneal and oral delivery, with distinct pharmacokinetic implications, and alcohol exposure paradigms ranged from acute single bolus to chronic Lieber–DeCarli feeding to voluntary two-bottle choice. Owing to this heterogeneity, a consistent dose–response relationship and an optimal therapeutic dose cannot be established from the available studies. Although DHM is generally reported to be well tolerated, systematic characterization of toxicity at the highest administered doses (up to 2 g/kg) is lacking and warrants dedicated investigation. Additionally, the timing of DHM administration relative to ethanol ranged from pre-treatment to concurrent treatment to post-exposure, and studies were conducted across multiple species and strains, including both mice and rats, with most studies restricted to male animals. This means that sex-related differences in DHM exposure and efficacy are largely uncharacterized across the included literature despite known sex differences in both ethanol metabolism and DHM pharmacokinetics [41]. Formulation represents another critical source of variability. Studies employed native DHM, enhanced delivery systems such as DHM-SEDDS and micellar DHM, and plant extracts. Among these sources of heterogeneity, the inclusion of plant extract-based studies is particularly consequential for interpreting the clinical findings.

Two extract types were used across the included studies: AGE and HDE. The degree to which findings from these studies can be attributed to DHM specifically varies considerably by source plant. DHM is a dominant bioactive flavonoid in *Ampelopsis grossedentata*, reaching percentages as high as 35% in dry extract [49], supporting its use as a reasonable surrogate for isolated DHM. In contrast, DHM constitutes less than 1% of HDE [50], making it difficult to attribute observed effects to DHM alone. Indeed, *Hovenia dulcis* contains additional flavonoids with known antioxidant and anti-inflammatory activity, including myricetin and quercetin, which may independently or synergistically contribute to the effects reported in HDE studies [50]. Therefore, the currently available clinical evidence should be interpreted as evidence for HDE rather than isolated DHM, and the extent to which DHM contributed to the observed clinical effects remains uncertain. This is particularly important given that both clinical studies utilized HDE, and neither reported concentrations of DHM in plasma [47,48]. Additionally, mechanistic insights from Kim et al. [47] provide further context for the observed clinical effects of HDE. The authors reported a significant correlation between total hangover severity scores and blood acetaldehyde levels, as well as with inflammatory cytokines including IL-6 and IL-10. Notably, CYP2E1 polymorphism emerged as a strong effect modifier of the relationship between hangover severity and these inflammatory markers. These findings suggest that the beneficial effects of HDE on hangover symptoms may be mediated, at least in part, through modulation of inflammatory homeostasis in response to alcohol exposure. Furthermore, the magnitude of this effect may vary depending on genetic variability in alcohol metabolism pathways, particularly CYP2E1 activity [47]. It is also worth noting that both trials were conducted in South Korea, where there is a longstanding cultural history of the use of *Hovenia dulcis* as an anti-hangover compound. Therefore, while the inclusion of these studies was justified, the extent to which their findings reflect DHM-specific pharmacology remains uncertain. Future clinical studies should prioritize well-characterized formulations containing isolated DHM.

The methodological heterogeneity across the included preclinical studies likely accounts, in part, for the inconsistency observed in several outcome measures, including BEC, ethanol-metabolizing enzyme activity, LORR, and voluntary alcohol consumption. When interpreting reductions in BEC, it is important to distinguish studies using controlled or forced ethanol exposure, in which ethanol intake was generally held constant, from voluntary-consumption paradigms, in which reduced drinking could itself lower BEC; consequently, only the former directly implicate a pharmacokinetic or metabolic effect of DHM. Reported reductions in BEC may also reflect delayed or reduced ethanol absorption, enhanced elimination, or a combination of both, and the included studies did not directly distinguish among these mechanisms. Because BEC was not consistently reported across studies, a complete cross-study extraction and comparison of these outcomes was not feasible.

Underlying many of these inconsistencies is the poor oral bioavailability of native DHM. Several studies reporting limited effects of native DHM used oral administration [33,38,39,44] whereas studies using intraperitoneal administration or enhanced formulations such as DHM-SEDDS and micellar DHM generally reported greater pharmacological activity. Oral DHM exhibits approximately 4% bioavailability in rats [41,51], and in mice oral administration yields roughly 7–24 times lower exposure than intraperitoneal administration depending on sex, with a plasma half-life of approximately 1.8–1.9 h following oral dosing [41]; inadequate systemic exposure may therefore explain some negative findings, particularly in studies administering native oral DHM with longer pre-treatment intervals. These studies also varied substantially in alcohol-exposure paradigm, species, sex, route of DHM and ethanol administration, timing of DHM relative to ethanol, and formulation [32,33,34,37,39,40,41,44,46]. Acute intoxication models assessing BEC or LORR following a single ethanol challenge may capture different pharmacological effects than chronic Lieber–DeCarli feeding or voluntary-consumption paradigms, which involve distinct biological processes including neuroadaptation, metabolic remodeling, and inflammatory responses. One particularly important methodological concern is that Shen et al. administered ethanol intraperitoneally [34], a route that bypasses first-pass metabolism and carries limited translational relevance compared with gastric administration; this is notable because Shen et al. provide much of the positive preclinical evidence for DHM’s neurobehavioral effects, which complicates the interpretation of those findings.

Beyond these in vivo considerations, the in vitro evidence base carries its own limitations. Because these data derive from a mixture of immortalized, carcinoma-derived (e.g., HepG2 and VL-17A), transformed epithelial (THLE-2), and primary cell systems, findings from transformed or cancer-derived lines may not generalize to non-transformed or primary cells. This is particularly relevant when interpreting mechanistic outcomes, as discrepancies were observed between model systems, including differing effects of DHM on ethanol-metabolizing enzymes in human carcinoma-derived cell lines compared with primary rat hepatocytes. In addition, one study (Ma et al. [27]) employed a markedly lower ethanol concentration (100 µM) than the other in vitro studies, which generally used approximately 50–300 mM, and its findings should therefore be interpreted with caution when compared with the broader in vitro evidence base. Taken together, these in vivo and in vitro inconsistencies likely reflect pharmacokinetic and methodological differences rather than a true inconsistency in DHM’s pharmacological activity.

DHM is a nutraceutical product currently available over the counter as an anti-hangover remedy and is generally recognized as safe (GRAS) by the FDA. While its safety profile appears favorable, clinical data remain limited, and rigorous evaluation of safety, pharmacokinetics, and drug–drug interactions is still needed [52]. The widespread availability of DHM-containing products, over 80 in the U.S. alone, highlights the potential for unsupervised use despite limited clinical validation [53]. Importantly, one of the biggest barriers to effective clinical translation is DHM’s poor aqueous solubility, driven by extensive intermolecular hydrogen bonding, which limits intestinal absorption and results in suboptimal pharmacokinetics, including low peak plasma concentrations and short half-life [51,54,55]. Addressing these pharmacokinetic limitations will therefore be a critical prerequisite for the clinical translation of DHM. Encouragingly, enhanced delivery formulations, including DHM-SEDDS and micellar DHM, demonstrated substantially improved relative oral bioavailability and more robust in vivo efficacy compared to native DHM [39,44], suggesting that formulation optimization may be key to unlocking DHM’s therapeutic potential.

Translation of these findings to humans remains challenging. Effective doses varied substantially across preclinical studies, ranging from 0.3 mg/kg to 2 g/kg, and direct extrapolation is complicated by species differences in absorption, metabolism, and bioavailability. Furthermore, the systemic exposure required to achieve therapeutic effects in humans remains unknown, particularly given the poor oral bioavailability of native DHM. Future studies should therefore incorporate pharmacokinetic-guided dose selection and establish exposure–response relationships to facilitate the rational translation of optimized DHM formulations into clinical trials.

## 5. Conclusions

Overall, current evidence supports a protective role for DHM in preclinical models of wholly alcohol-attributed conditions, particularly through reductions in steatosis, oxidative stress, inflammation, and mitochondrial dysfunction. In contrast, findings related to alcohol-associated neurobehavioral outcomes remain inconsistent, likely reflecting methodological heterogeneity across studies. However, clinical evidence remains limited and is derived exclusively from studies using HDE, which do not directly represent the pharmacological activity or clinical efficacy of isolated DHM. Consequently, there is currently no clinical evidence supporting DHM for the treatment of AUD, ALD, alcohol withdrawal, or alcohol-related neurological conditions. Although DHM remains a promising candidate given its favorable safety profile and robust preclinical efficacy, notably, enhanced delivery systems such as DHM-SEDDS and micellar DHM, as well as parenteral administration, consistently outperformed native oral DHM in preclinical models, indicating that formulation optimization will likely be central to translating DHM into effective clinical interventions. Future studies should prioritize well-characterized formulations containing isolated DHM and evaluate their efficacy in adequately powered clinical trials targeting AUD, FASDs, and ALD.

## Figures and Tables

**Table 1:** Characteristics of in vitro studies included.

Author, Year	Model	EtOH Dose	DHM Dose	Treatment Time	Outcomes Measured
Ma et al. (2025) [27] ^a^	AML-12 cells	100 μM EtOH	50 μM DHM	36 h (concurrent DHM + EtOH)	cell viability and oxidative stress markers
				48 h (concurrent DHM + EtOH)	lipid accumulation
	THLE2 cells	100 μM EtOH	50 μM DHM	48 h (concurrent DHM + EtOH)	mitochondrial membrane potential, apoptosis, and senesce-related proteins
Luo et al. (2025) [28]	THLE2 cells	100 mM EtOH	50 μg/mL AGE	24 h (concurrent DHM + EtOH)	oxidative stress markers
deGodoi et al. (2023) [29]	SH-SY5Y cells	4 mg/mL EtOH	0.125 mg/mL, 0.25 mg/mL, or 0.5 mg/mL HDE	3 h HDE 3 h EtOH (DHM pre-treatment)	cell viability and oxidative stress markers
Wang et al. (2023) [30]	AML-12 cells	400 mmol/L EtOH	25 μmol/L, 50 μmol/L, or 100 μmol/L DHM	24 h DHM 24 h EtOH (DHM pre-treatment)	cell viability, liver injury enzymes, oxidative stress/antioxidant markers, EtOH metabolism markers, and gut barrier markers
	IEC-6 cells	400 mmol/L EtOH	25 μmol/L, 50 μmol/L, or 100 μmol/L DHM	24 h (DHM pre-treatment)	cell viability, liver injury enzymes, oxidative stress/antioxidant markers, EtOH metabolism markers, and gut barrier markers
		400 mmol/L EtOH	50 μmol/L DHM	12–36 h DHM 24 h EtOH (DHM pre-treatment)	transepithelial electrical resistance
			50 μmol/L DHM	12–48 h DHM 24 h EtOH (DHM pre-treatment)	paracellular permeability
Getachew et al. (2022) [31]	SH-SY5Y cells	500 mM EtOH	0.01 nM, 0.1 nM, 1 nM, 10 nM, or 100 nM DHM (Added one hour prior to EtOH)	24 h (concurrent DHM + EtOH)	cell viability
Silva et al. (2020) [32] ^†^	HepG2 cells	50 mM, 100 mM, or 200 mM EtOH	0.1 μM to 50 μM DHM (2.5 μM or 5 μM DHM used most frequently)	2–72 h DHM (concurrent DHM + EtOH)	lipid accumulation, apoptosis and inflammatory markers, cell viability, EtOH metabolism markers, and oxidative stress markers
	VL-17A cells	50 mM, 100 mM, or 200 mM EtOH	0.1 μM to 50 μM DHM (2.5 μM or 5 μM DHM used most frequently)	2–72 h DHM (concurrent DHM + EtOH)	lipid accumulation, apoptosis and inflammatory markers, cell viability, EtOH metabolism markers, and oxidative stress markers
Skotnicová et al. (2020) [33]	Primary rat hepatocytes isolated following in vivo EtOH exposure (40% EtOH gavage for 4 days; 20% EtOH as sole drinking source for 2 days)	300 mM EtOH	1 μM, 10 μM, 50 μM, or 100 μM DHM	15 h (concurrent DHM + EtOH)	cell viability, liver injury enzymes, and oxidative stress markers
		EtOH concentration(s) not specified	10 μM, 50 μM, or 100 μM DHM	15 h (concurrent DHM + EtOH)	EtOH metabolism markers
Shen et al. (2012) [34]	Dissociated primary hippocampal neurons from E18 rats	60 mM EtOH	0.1, 0.3, 1, 3, 10, or 30 μM DHM	30 min EtOH exposure, 24 h withdrawal before recording	patch-clamp recordings/GABA_A_R potentiation, mIPSCs, and tonic currents
	Hippocampal slices (Dentate granule cells)	60 mM EtOH	0.1, 0.3, 1, 3, 10, or 30 μM DHM	acute application during recording	patch-clamp recordings/GABA_A_R potentiation, mIPSCs, and tonic currents

AML-12: Alpha Mouse Liver 12; DHM: dihydromyricetin; E18: embryonic day 18; EtOH: ethanol; GABA_A_R: gamma-aminobutyric acid type A receptor; HepG2: human hepatocellular carcinoma cell line; IEC-6: intestinal epithelial cell line 6; mIPSCs: miniature inhibitory postsynaptic currents; SH-SY5Y: human neuroblastoma cell line; THLE2: transformed human liver epithelial 2; VL-17A: HepG2-derived human hepatocellular line constitutively expressing alcohol dehydrogenase and CYP2E1. ^†^ Due to the wide range of EtOH and DHM concentrations and exposure paradigms used across assays in Silva et al.’s study, dosing information is summarized rather than listed for each individual experiment. In contrast, other studies report discrete dose–outcome pairings that are presented explicitly. ^a^ An ethanol concentration of 100 µM is reported as stated in the original study [27] and is substantially lower than the millimolar concentrations used in the other included in vitro studies.

**Table 2:** Characteristics of in vivo studies included.

Author, Year	Model	Alcohol Paradigm	DHM Dose	Outcomes Measured
Ma et al. (2025) [27]	Male C57BL/6 mice	LDC diet containing 5% (*v*/*v*) EtOH—7 weeks	75 mg/kg or 150 mg/kg DHM (p.o., frequency not specified)	liver injury enzymes, inflammatory/oxidative markers, hepatic steatosis, senescence and cell cycle-related proteins (miR-155-5p, SIRT1, and VDAC1), and serum metabolites
Luo et al. (2025) [28]	Male C57BL/6 mice	LDC diet containing 5% (*v*/*v*) EtOH—7 weeks	150 mg/kg or 300 mg/kg AGE (p.o., once daily)	liver injury enzymes, inflammatory/oxidative markers, hepatic steatosis, and mitochondrial function markers (YTHDF2, PGC-1α, and SIRT3)
Sun et al. (2025) [35]	Male SPF ICR mice	Single bolus, 7.5 mL/kg EtOH, p.o.	0.625 g/kg, 1.25 g/kg, or 2.5 g/kg AGE (p.o., single dose prior to EtOH administration)	duodenal injury markers, amino acid metabolism, and apoptosis-related proteins (TNFα, FADD, caspase-8, IL-17, and MAPK)
Qiu et al. (2024) [36]	Male C57BL/6 mice	LDC diet containing 5% (*v*/*v*) EtOH—7 weeks	150 mg/kg or 300 mg/kg AGE (ROA not specified, frequency not specified)	liver injury enzymes, inflammatory/oxidative markers, hepatic steatosis, TLR4-pathway proteins (TLR4, NFκB, and MLKL), and gut barrier markers (ZO-1 and occludin)
Niiya et al. (2024) [37]	Male Wistar rats	Single bolus, 20% EtOH (1.2 g/kg), p.o.	1 or 2 g/kg HD water extract (p.o., 30 min prior to EtOH administration)	BEC and EtOH metabolism markers
Janilkarn-Urena et al. (2023) [38]	Female C57BL/6 mice	LDC diet containing 5.5% (*v*/*v*) EtOH—5 weeks	6 mg/mL DHM (p.o., once daily)	liver injury enzymes, inflammatory/oxidative markers, hepatic steatosis, and lipophagy-related proteins (p62, LC3B, and PLIN-1)
Dong et al. (2023) [39]	Male Kunming mice	Single bolus, 56% EtOH (0.18 mL/10 g), p.o.	100 mg/kg DHM (p.o., one hour prior to EtOH administration)	loss of righting reflex and BEC
		Single bolus, 56% EtOH (0.14 mL/10 g), p.o.	100 mg/kg DHM (p.o., once daily for 7 days, before EtOH administration)	EtOH metabolism markers
		Single bolus, 0.1 mL/10 g, p.o.	100 mg/kg DHM (p.o., one hour prior to EtOH administration)	gastric mucosal injury markers
Wu et al. (2022) [40]	Male C57BL/6 mice	NIAAA model (LDC diet containing 5% (*v*/*v*) 10 days plus binge)	250 mg/kg AGE 250 mg/kg Ampelopsin (p.o., once daily)	liver injury enzymes, hepatic steatosis, and EtOH metabolism markers
Carry et al. (2021) [41]	Male and female C57BL/6 mice	Single bolus, 5 g/kg EtOH, p.o.	50 mg/kg DHM (i.p., single dose)	loss of righting reflex
Silva et al. (2021) [42]	Male C57BL/6 mice	Single bottle access to 30% EtOH for 8 weeks, p.o.	5 mg/kg DHM (i.p., five days/week)	metabolic signaling markers (AMPK, SIRT1, and PGC-1α) and mitochondrial function markers (TFAM, complex proteins, and ATP)
Silva et al. (2021) [43]	Male and female C57BL/6 mice	TBC (10% EtOH, p.o.)	10 mg/kg DHM (i.p., once daily)	EtOH intake
Ye et al. (2021) [44]	Male Sprague-Dawley rats	Single bolus, 70% EtOH (0.16 mL/10 g), p.o.	100 mg/kg DHM (p.o., 2 h prior to EtOH administration)	BEC
	Male Kunming mice	Single bolus, 70% EtOH (0.16 mL/10 g), p.o.	100 mg/kg DHM (p.o., 2 h prior to EtOH administration)	loss of righting reflex
	Male C57BL/6 mice	Single bolus, 50% EtOH (0.1 mL/10 g), p.o.	100 mg/kg DHM (p.o., one hour prior to EtOH administration)	gastric mucosal injury markers
		Triple bolus (each 12 h apart), 50% EtOH (0.1 mL/10 g), p.o.	100 mg/kg DHM (p.o., once daily, six days)	liver injury enzymes and oxidative markers
Skotnicová et al. (2020) [33]	Male Wistar rats	Single bolus (second bolus at 24 h in subset), 40% EtOH, p.o.	10 mg/kg DHM (p.o., co-administered with EtOH)	EtOH metabolism markers
Silva et al. (2020) [32]	Male C57BL/6 mice	Single bottle access to 30% EtOH for 8 weeks, p.o.	5 mg/kg or 10 mg/kg DHM (i.p., once daily)	liver injury enzymes, inflammatory/oxidative markers, and hepatic steatosis, metabolic signaling markers (AMPK, SREBP1, CPT1a, and Nrf2)
		3.5 g/kg EtOH i.p	5 mg/kg DHM (i.p., co-administered with EtOH)	EtOH metabolism markers
Qiu et al. (2017) [45]	Male C57BL/6 mice	LDC diet containing 5% (*v*/*v*) EtOH—7 weeks	75 mg/kg or 150 mg/kg DHM (p.o., once daily)	liver injury enzymes, inflammatory/oxidative markers (Nrf2 and Keap1), hepatic steatosis, and autophagy markers (p62 and LC3B)
Liang et al. (2014) [46]	First-pregnancy Sprague-Dawley rats	Five boluses over course of pregnancy, 1.5 g/kg, 2.5 g/kg, or 5 g/kg EtOH, p.o.	1 mg/kg DHM (p.o., five administrations over course of pregnancy)	offspring developmental outcomes, loss of righting reflex, seizure susceptibility, and GABA_A_R function
Shen et al. (2012) [34]	Male Sprague Dawley rats	Single bolus, 3 g/kg or 4 g/kg EtOH, i.p.	0.3 mg/kg, 0.5 mg/kg, 1 mg/kg, or 10 mg/kg DHM (i.p., co-administered with EtOH)	loss of righting reflex, BEC, and withdrawal behaviors
		TBC (5 g/kg EtOH, p.o.)	0.05 mg/mL DHM (p.o., administered in drinking water)	EtOH intake and BEC

AGE: *Ampelopsis grossedentata* extract; AMPK: AMP-activated protein kinase; ATP: adenosine triphosphate; BEC: blood ethanol concentration; CPT1a: carnitine palmitoyltransferase 1a; DHM: dihydromyricetin; EtOH: ethanol; FADD: Fas-associated death domain; GABA_A_R: gamma-aminobutyric acid type A receptor; HD: *Hovenia dulcis*; i.p.: intraperitoneal; ICR: Institute of Cancer Research; IL-17: interleukin-17; Keap1: Kelch-like ECH-associated protein 1; LC3B: microtubule-associated protein 1 light chain 3 beta; LDC: Lieber–DeCarli; MAPK: mitogen-activated protein kinase; MLKL: mixed lineage kinase domain-like pseudokinase; NFκB: nuclear factor kappa B; NIAAA: National Institute on Alcohol Abuse and Alcoholism; Nrf2: nuclear factor erythroid 2-related factor 2; p.o.: per os; p62: sequestosome 1; PGC-1α: peroxisome proliferator-activated receptor gamma coactivator 1-alpha; PLIN-1: perilipin-1; ROA: route of administration; SIRT: sirtuin; SPF: specific pathogen-free; SREBP1: sterol regulatory element-binding protein 1; TBC: two-bottle choice; TFAM: mitochondrial transcription factor A; TLR4: toll-like receptor 4; TNFα: tumor necrosis factor alpha; VDAC1: voltage-dependent anion channel 1; YTHDF2: YTH N6-methyladenosine RNA binding protein 2; ZO-1: zonula occludens-1.

## Data Availability

No new data were created or analyzed in this study. Data sharing is not applicable to this article.

## Abbreviations

The following abbreviations are used in this manuscript:

ADH	Alcohol dehydrogenase
AGE	*Ampelopsis grossedentata* extract
ALD	Alcohol-associated liver disease
ALDH	Aldehyde dehydrogenase
ALT	Alanine aminotransferase
AMPK	AMP-activated protein kinase
AST	Aspartate aminotransferase
AUD	Alcohol use disorder
CNS	Central nervous system
CPT1a	Carnitine palmitoyltransferase-1a
CYP2e1	Cytochrome P450 2E1
DHM	Dihydromyricetin
DMY-Ms	Micellar formulation of dihydromyricetin
DHM-SEDDS	Self-emulsifying drug delivery system of dihydromyricetin
EtOH	Ethanol
ERK	Extracellular signal-regulated kinase
FADD	Fas-associated death domain
FASDs	Fetal alcohol spectrum disorders
FDA	Food and Drug Administration
GABA_A_R	Gamma-aminobutyric acid type A receptor
GM-CSF	Granulocyte–macrophage colony-stimulating factor
GSH	Glutathione
H&E	Hematoxylin and eosin
HDE	*Hovenia dulcis* extract
HO-1	Heme oxygenase-1
ICAM-1	Intercellular adhesion molecule 1
IEC-6	Intestinal epithelial cell line 6
IFN	Interferon
IL	Interleukin
i.p.	Intraperitoneal
JNK	c-Jun *N*-terminal kinase
Keap1	Kelch-like ECH-associated protein 1
LBP	Lipopolysaccharide-binding protein
LC3B	Microtubule-associated protein 1 light chain 3 beta
LDC	Lieber–DeCarli
LDH	Lactate dehydrogenase
LORR	Loss of righting reflex
MAPK	Mitogen-activated protein kinase
M-CSF	Macrophage colony-stimulating factor
MDA	Malondialdehyde
MeSH	Medical subject headings
miR	MicroRNA
MLKL	Mixed lineage kinase domain-like pseudokinase
mRNA	Messenger ribonucleic acid
mtDNA	Mitochondrial DNA
MyD88	Myeloid differentiation primary response 88
NAD^+^	Nicotinamide adenine dinucleotide (oxidized form)
NADH	Nicotinamide adenine dinucleotide (reduced form)
NF-κB	Nuclear factor kappa B
nDNA	Nuclear DNA
NIAAA	National Institute on Alcohol Abuse and Alcoholism
Nrf2	Nuclear factor erythroid 2-related factor 2
p.o.	Per os (oral administration)
PGC-1α	Peroxisome proliferator-activated receptor gamma coactivator 1-alpha
PLIN-1	Perilipin-1
PRISMA	Preferred Reporting Items for Systematic Reviews and Meta-Analyses
PTZ	Pentylenetetrazol
ROS	Reactive oxygen species
SIRT	Sirtuin
SOD	Superoxide dismutase
SREBP-1	Sterol regulatory element-binding protein 1
TFAM	Mitochondrial transcription factor A
TIMP1	Tissue inhibitor of metalloproteinases 1
TLR	Toll-like receptor
TNF-α	Tumor necrosis factor alpha
TOMM20	Translocase of the outer mitochondrial membrane 20
VDAC1	Voltage-dependent anion channel 1
WHO	World Health Organization
YTHDF2	YTH N6-methyladenosine RNA binding protein 2
ZO-1	Zonula occludens-1
